# Telephone consultations with otolaryngology – head and neck surgery reduced emergency visits and specialty consultations in northern Alberta

**DOI:** 10.1186/s40463-020-00439-0

**Published:** 2020-06-22

**Authors:** Peter George Jaminal Tian, Dean Eurich, Hadi Seikaly, Douglas Boisvert, John Montpetit, Jeffrey Harris

**Affiliations:** 1 Department of Family Medicine, University of Alberta, Edmonton, Canada; 2 School of Public Health, University of Alberta, Edmonton, Canada; 3 Division of Otolaryngology, Department of Surgery, University of Alberta, Edmonton, Canada; 4 RAAPID North, Alberta Health Services, Edmonton, Canada; 5 Poison and Drug Information Service and RAAPID, Alberta Health Services, Calgary, Canada

## Abstract

**Background:**

RAAPID (Referral, Access, Advice, Placement, Information, and Destination) is a 24-h call center in Alberta, Canada, facilitating urgent telephone consultations between physicians and specialists. We evaluated the extent to which RAAPID calls to Otolaryngology-Head and Neck Surgery (OHNS) reduced visits to the emergency department and specialty clinics.

**Methods:**

This was a cross-sectional study evaluating all telephone consultations to OHNS from physicians in northern Alberta between 2013 and 2014 (T1) (where consultations by residents occurred) and 2015 to 2017 (T2) (where consultations were done by consultants during office hours and residents during after hours). Outcomes of the calls included medical advice, specialty clinic referrals, and emergency department (ED) referrals. Differences in the reduction of ED visits and costs, overall as well as in T1 and T2, were assessed using multivariate logistic regression.

**Results:**

Overall, 62.3% (1064/1709) of telephone consultations reduced ED visits consisting of advice being provided (*n* = 884; 83.1%) and referral to specialty clinics (*n* = 180; 16.9%). The adjusted odds ratio of calls reducing emergency visits in T2 as compared to T1 was 2.47 (95% CI 1.99 to 3.08). The adjusted odds ratio of reducing ED visits during office hours compared to after-hours 2.54 (95% CI 1.77–3.64). The estimated direct costs avoided from ED visits in T1 and T2 were $42,224.22 and $114,393.86, respectively.

**Conclusion:**

RAAPID telephone consultations to OHNS were effective in reducing ED visits and healthcare costs. This model should be considered in other areas to improve efficiencies within the health system.

## Introduction

Telephone consultations as part of an eHealth approach which allow healthcare providers to access specialists or specialty teams are increasingly being used in Canada. For example, R.A.C.E. (Rapid Access to Consultative Expertise) is a telephone hotline for family physicians and nurse practitioners in Vancouver, British Columbia, Canada. The program reported that 32% of calls avoided an emergency department (ED) visit and 60% of calls avoided the need for specialist visits. These outcomes translated to an estimated of up to $200 of cost avoidance per call [1]. Other Canadian provinces have their own versions of telephone consultations. However, the programs’ outcomes have not been reported. In the U.S., numerous programs also exist. For example, the Massachusetts Child Psychiatry Access provides a hotline to pediatric primary care clinicians for consults to a child psychiatry team [2]. The authors reported that 24% of consults resulted in the primary care clinicians retaining care for the patients. In Italy, a service to access cardiologists, dermatologists, and diabetologists was provided to general practitioners. This resulted in the avoidance of ED visits, hospitalizations, or in-clinic consultations in 77% of calls [3].

Alberta, Canada, has its own program called RAAPID (Referral, Access, Advice, Placement, Information, and Destination) which is a 24-h call center. RAAPID facilitates urgent telephone consultations between Alberta’s physicians with specialists in tertiary care centers. This urgent telephone consultation allows patients to be cared for in their own communities, referred to outpatient clinics, or dispatched to emergency departments when needed. Among RAAPID’s telephone consultations are those to Otolaryngology – Head and Neck Surgery (OHNS), mainly in the University of Alberta Hospital, Edmonton, Alberta, Canada. Physicians in rural communities dealing with serious airway infections, for example, may call RAAPID to be connected with on-call OHNS staff for medical advice. Whether this service is effective in reducing ED visits is unclear. Thus, we aimed to evaluate whether RAAPID-North’s calls to OHNS between 2013 and 2017 reduced visits to the ED, visits to specialty clinics, and healthcare costs.

## Methods

**Fig. 1:**
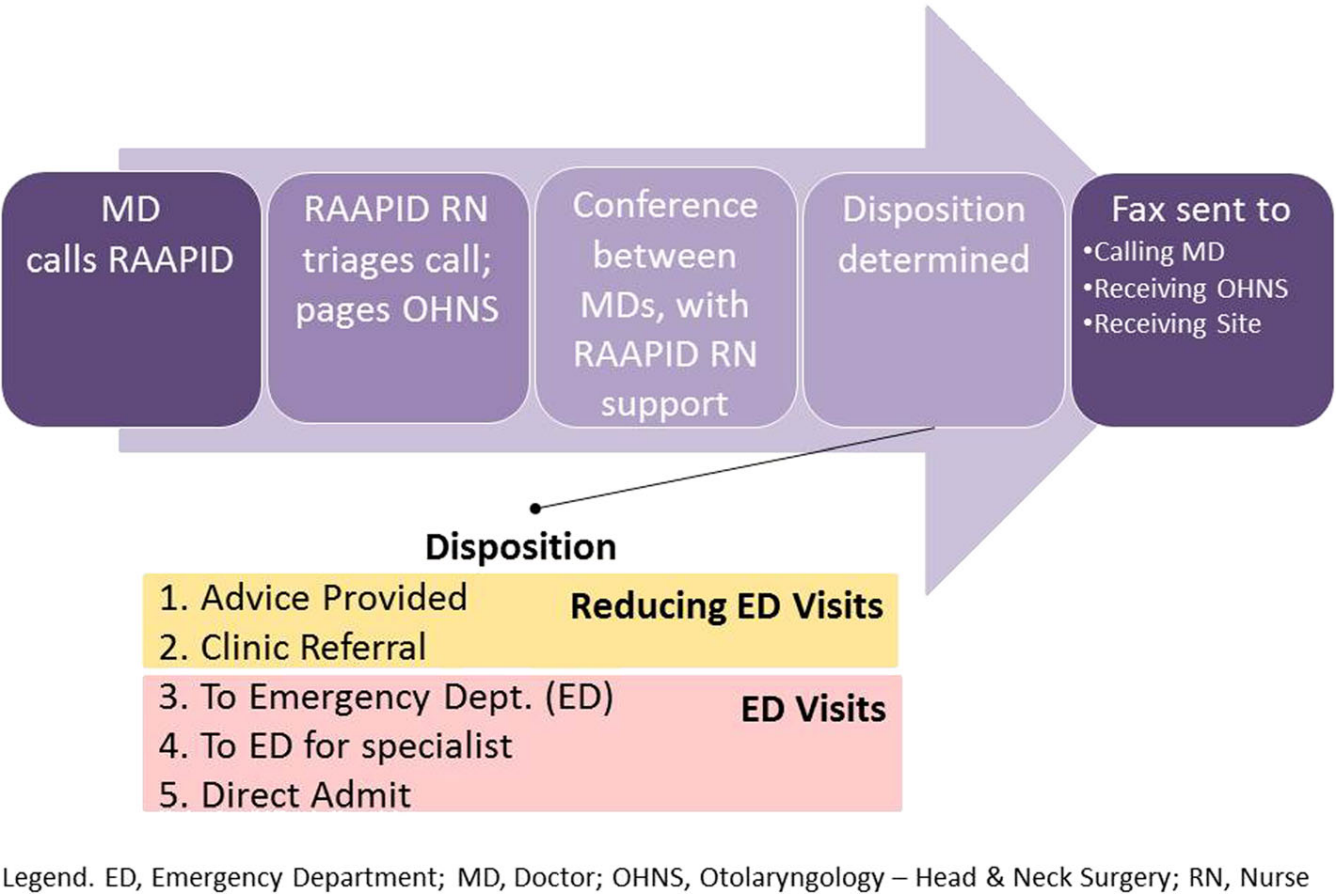
The RAAPID Referral Process

This was a cross-sectional study with approval from the University of Alberta Health Research Ethics Board (Study ID No. Pro00081649). Between 2013 to 2017, all calls to OHNS in the RAAPID-North Program were evaluated. The RAAPID-North call centre facilitates all urgent calls from physicians located north of the city of Red Deer (Alberta, Canada) which is located between Alberta’s two major urban centres (Edmonton and Calgary). Briefly, the telephone consultation process is started when the referring physician calls the 24-h RAAPID call centre. A nurse receives and triages the call, pages the OHNS staff to arrange a teleconference between the physicians, and receives and executes the disposition [4]. Following each call, detailed information is entered into an administrative database to document the service and includes: date and time of call; patient age and sex; physician caller’s site; receiving physician’s specialty and site (the University of Alberta Hospital in 98% of calls); response time of the OHNS staff to the page from RAAPID; disposition (i.e., action advised) after the telephone consultation; and time the call was cleared, defined as the time elapsed when the call was received by RAAPID to the time the consultation was completed. The time to clear a call represented the total duration required to address the calling physician’s concerns, inclusive of time elapsed for administrative support, response time of OHNS staff, and consultation time. See Fig. 1 below. The RAAPID program provided these data to the authors after the latter obtained approval from Alberta Health Services.

Figure adapted and modified from: Montpetit J, Burke D, Carlson K. DTN – Interfacing with RAAPID. Quality Improvement and Clinical Research – Alberta Stroke Program, University of Calgary, Calgary, AB. 2017 [cited 2017 Jul 28].]

### Outcomes

Our main outcome of interest was reduction of ED visits after the telephone consultations. Each RAAPID call received by a consultant or resident is associated with a final disposition of one of the following: (1) medical advice only to the physician caller, (2) referral to an outpatient specialist clinic, and (3) medical advice to proceed to the ED. Consultations resulting only in advice to the physician caller or advising referrals to an outpatient specialty clinic were classified as consultations reducing ED visits. This classification is justified as the calling physicians have no other options available to assist in the management of their patients in the province. As a result, directing patients to an ED may be the only avenue available to these patients to obtain the medical assessments/advice required in the absence of the RAAPID program. We categorized telephone consultations advising referrals to the ED or advising direct admissions as consultations resulting in ED visits, irrespective of whether this actually occurred. Other studies have used dispositions or plans after the telephone consultations to estimate the prevention of ED visits or specialty consults [1, 3, 5–7].

### Statistical methods

Descriptive statistics were used to describe the characteristics of the patients and calls. To evaluate the overall impact of the RAAPID program, the entire time period between 2013 to 2017 was used. We divided the period into two separate time frames of analysis: January 1, 2013 to December 31, 2014 (Time 1, T1) and June 1, 2015 to May 31, 2017 (Time 2, T2). The rationale for having two evaluation periods was the implementation of a procedural change in 2015. Before January 2015, RAAPID calls were initially taken by OHNS residents. However, in January 2015, a procedural change routed all office-hour (i.e., 0900H to 1659H Monday to Friday) RAAPID consults directly to the OHNS consultants while after-hour calls were still directed to residents. For analyses, we evaluated the outcomes of the two time frames combined and also compared the outcomes before (T1) and after (T2) the procedural change, as well as for office-hour and after-hour consultations. To allow sufficient time for the new procedures to be implemented in T2, a washout period of 5 months (Jan 1, 2015 to May 31, 2015) was used and excluded from all analyses as it is expected that the program was less efficient as new procedures were implemented. Logistic regression was used to estimate the odds ratios of reducing ED visits between T1 and T2 after adjusting for the patients’ sex and age. Stata version 15.1 SE (StataCorp, College Station, Texas) was used for all analyses.

With respect to cost avoidance, we defined cost avoidance as the estimated expense avoided from consultation fees and average ED visit cost in Alberta. The cost model assumed the following: (a) all the patients would have been referred to the ED had there been no RAAPID telephone consultations; (b) the calling physician would not bill an additional fee for the RAAPID call because he would have billed for the initial consultation of the patient; (c) the OHNS consultant called would bill for the RAAPID call; (d) the OHNS resident called would not bill for the RAAPID call as they do not receive service fees in Alberta; (e) additional costs from diagnostic and management procedures would remain equivalent had the RAAPID call not occurred.

**Fig. 2:**
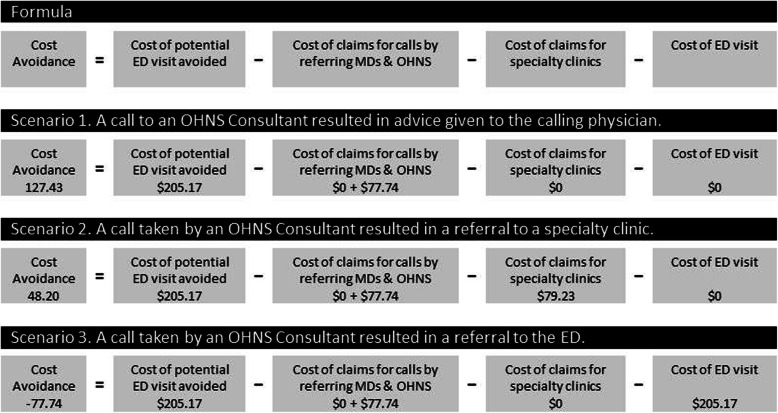
Formula for estimating cost avoidance and sample scenarios. See Additional file 1 for detailed computations and reference values

The cost model entails that had there been no RAAPID consultation, the patient would have been sent to the ED and the average of cost of an ED visit would have been incurred by the healthcare system. See Formula (Fig. 2). Here are three sample scenarios.

#### Scenario 1

When a RAAPID consultation to an OHNS consultant resulted in a disposition of advice given to the calling physician, the healthcare system would have avoided the cost of an ED visit, minus the cost of physician claims for the calls.

#### Scenario 2

When the consultation to an OHNS consultant resulted in a disposition of being referred to a specialty clinic, the healthcare system would have avoided the cost of an ED visit minus the cost of the physician claims for the calls and minus the physician claim for the specialty clinic.

#### Scenario 3

When a RAAPID consultation to an OHNS consultant resulted in a disposition of being referred to the ED, the potential avoided cost of an ED visit is cancelled by the referral for an ED visit. Moreover, in this scenario, the healthcare system would have incurred the cost of physician claims for the calls.

See Additional file 1 for the detailed costs estimates and reference values across 12 scenarios. This cost model only considers the average cost of an ED visit for OHNS patients. The model underestimates the costs from additional fees associated with OHNS ED consult and incidental procedures (e.g., endoscopy). Moreover, indirect costs (e.g., patient travel, time off work, administrative cost of the ED program and RAAPID program, ground and air emergency transportation) were not included in the model because of lack of data.

## Results

Between 2013 to 2017, there were 1709 RAAPID calls to OHNS from the RAAPID-North program. There were 474 (27.7%) calls in T1 and 1235 (72.3%) calls in T2. This represented an increase of 261% from T1 to T2. Out of the total calls, 45.6% of calls occurred during office hours on weekdays, 29.5% during after-office hours on weekdays, and 24.9% during weekends (Table 1). Eleven calls were excluded because of the uncertainty of the dispositions.

**Table 1:** Characteristics patients, calls, and callers by time frame

	Overall (***n*** = 1709) Mean (SD) or n (%)	T1 (***n*** = 474) Mean (SD) or n (%)	T2 (***n*** = 1235) Mean (SD) or n (%)	***P***-value^a^
Patient Characteristics				
Age in years– Overall	45.6 (21.9)	41.3 (25.4)	47.2 (20.2)	< 0.001
Sex – male	967 (56.6)	277 (58.4)	690 (55.9)	0.529
Characteristics of Calls				
Calls during office hours	779 (100)	169 (35.6)	610 (78.3)	< 0.001
Calls during after hours - weekday	504 (100)	164 (32.5)	340 (67.5)	< 0.001
Calls during after hours - weekend	426 (100)	141 (33.1)	285 (66.9)	< 0.001
Response Time (minutes)				
Overall	18.9 (34.6)	11.4 (20.7)	21.8 (38.4)	< 0.001
Calls during office hours	26.0 (42.9)	12.7 (23.0)	29.9 (46.5)	< 0.001
Calls during after hours - weekday	15.0 (26.6)	11.3 (16.5)	16.7 (30.0)	0.036
Calls during after hours - weekend	11.2 (22.7)	9.9 (22.1)	11.8 (23.0)	0.425
Time to clear call (hours)				
Overall	0.9 (1.2)	0.9 (1.6)	0.8 (1.0)	0.169
Calls during office hours	0.9 (1.1)	0.8 (1.2)	1.0 (1.0)	0.103
Calls during after hours - weekday	0.9 (1.5)	1.1 (2.1)	0.8 (1.1)	0.025
Calls during after hours - weekend	0.7 (1.0)	0.9 (1.2)	0.6 (0.9)	0.023
Distance of caller to University of Alberta Hospital (kms)				
0–50 km	896 (52.4)	166 (35.0)	730 (59.1)	< 0.001
51–100	166 (9.7)	68 (14.4)	98 (7.9)	0.180
101–150	182 (10.7)	64 (13.5)	118 (9.6)	0.422
151–200	110 (6.4)	42 (8.9)	68 (5.5)	0.491
201–250	152 (8.9)	66 (14.0)	86 (7.0)	0.155
251–300	91 (5.3)	39 (8.2)	52 (4.2)	0.423
> 300	112 (6.6)	29 (6.1)	83 (6.7)	0.911

^a^for comparison of T1 to T2

The average age of patients was 45.6 years (SD 21.9 years). The age of the patients ranged from 0 to 100 years with 73% (*n* = 1249) of patients belonging to the 21–70 age categories. Patients in T1 had a slightly lower mean age than T2 (41.3 vs 47.2 years, *p* < 0.001) (Table 1). Overall, 43.4% of patients were females, with no difference between T1 and T2 (*p* = 0.346) (Table 1).

After receiving the page from RAAPID, the average response time was 18.9 min (SD 34.6) for the specialist/resident and 50.4 min (SD: 72.5 min) to clear a call. Majority of calls (*n* = 1281, 75%) were cleared within an hour and 90% (*n* = 1546) within 2 h. The time to response and time to clear during office hours was quicker than after hours in both T1 and T2 (Table 1).

As expected, 97.1% (1660/1709) of the callers were from Alberta, with the rest from Northwest Territories (*n* = 39, 2.3%) of which the University of Alberta Hospital is a service facility, Saskatchewan (*n* = 7, 0.4%), Nunavut (*n* = 2, 0.1%), and British Columbia (*n* = 1, 0.1%). 52% of calls came from sites within 50 km to the University of Alberta Hospital while 38% of the calls were from sites farther than 100 km (Table 2). Most of the physician callers (98.3%; 1680/1709) were connected to OHNS in the University of Alberta Hospital. The rest of the callers were routed to other facilities, mostly in Edmonton.

**Table 2:** Dispositions of calls by time frame

	T1	T2	Overall
**Overall**	**474 (100%)**	**1235 (100%)**	**1709 (100%)**
**1. Reducing ED Visits – Overall**	**217 (45.8%)**	**847 (68.6%)**	**1064 (62.3%)**
a. Advice Provided	188 (86.6%)	696 (82.2%)	884 (83.1%)
b. Referral to Clinic	29 (13.4%)	151 (17.8%)	180 (16.9%)
**2. ED Visit/Direct Admission Recommended**	**257(54.2%)**	**388 (31.4%)**	**645 (37.7%)**
Crude OR – T2 compared to T1;	2.58 (95% CI: 2.08 to 3.21)		–
Adjusted OR	2.48 (95% CI: 1.99.0 to 3.08)		
**Calls- Office Hours (*n*** **= 779)**	**169 (35.7%)**	**610 (49.4%)**	**779 (45.6%)**
**1. Reducing ED Visits**	**92 (54.4%)**	**465 (76.2%)**	**557 (71.5%)**
a. Advice Provided	80 (87.0%)	385 (79.4%)	465 (83.5%)
b. Referral to Clinic	12 (13.0%)	80 (16.5%)	92 (16.5%)
**2. ED Visit/Direct Admission Recommended**	**77 (45.6%)**	**145 (23.8%)**	**222 (28.5%)**
Crude OR – T2/T1 Adjusted OR	2.68 (95% CI: 1.88 to 3.83) 2.54 (95% CI: 1.77 to 3.64)		–
**Calls- After-Office Hours(*n*** **= 930)**	**305 (64.3%)**	**625 (50.6%)**	**930 (54.4%)**
**1. Reducing ED Visits**	**125 (41.0%)**	**382 (61.1%)**	**507 (54.5%)**
a. Advice Provided	108 (86.4%)	311 (81.4%)	419 (82.6%)
b. Referral to Clinic	17 (13.6%)	71 (18.6%)	88 (17.4%)
**2. ED Visit/Direct Admission Recommended**	**180 (59.0%)**	**243 (38.9%)**	**423 (45.5%)**
Crude OR – T2/T1	2.26 (95% CI: 1.71 to 2.99)		–
Adjusted OR	2.19 (95% CI: 1.66 to 2.91)		

Regarding the primary endpoint, 62.3% (1064/1709) of RAAPID calls resulted in the reduction of ED visits. Of these calls, 83% (*n* = 884) of calls resulted in advice being provided to the calling physician, allowing for care delivery in the community while 17% (*n* = 180) of calls resulted in a referral to a specialists clinic, allowing for outpatient consultations. Of the remaining calls, 37.7% (*n* = 645) of calls resulted in a recommendation of an ED visit or direct admission to hospital. A slightly higher reduction in ED visits was noted during normal office hours (*n* = 557, 71.5%) compared to after office hours calls (*n* = 507, 54.5%) (*p* < 0.001).

Comparing T1 and T2, more calls in T2 resulted in a reduction of ED visits (68.6%) than in T1 (45.8%): adjusted odds ratio (OR) 2.48 (95% CI: 1.99 to3.08). This trend was consistently observed in both calls during office-hour and after-hours calls during the weekday or weekend. Moreover, more calls completed during office hours resulted in a reduction of ED visits than during after-hours irrespective of time period evaluated. See Table 2.

With respect to cost avoidance, in total, the cost avoided from the telephone consultations was estimated to be $156,618.08, with more cost avoided in T2 ($114,393.86) than T1 ($42,224.22) which is a reflection of both the increased number of calls and higher percentage of calls with reducing ED visits. In T1, more cost was avoided during after-office hours ($24,299.34) than during office hours ($17,924.88). In T2, the cost avoided during after-office hours ($72,749.61) was also more than during office hours ($41,644.25). See Table 3. Overall, the average cost avoided per consultation was $91.64, with T2 ($92.63) slightly higher than T1 ($89.08).

**Table 3:** Cost avoided

(1)	Cost Avoided per Call^a^ (2)	Cost Avoided (1)*(2) (Mean Cost Avoided per Call)^b^
**Time 1: Office HoursT(*n* = 169)**		**$17,924.88** **($106.06)**
Office hours: advice (*n* = 80)	205.17	16,413.60
Office hours: referral to clinic (*n* = 12)	125.94	1511.28
Office hours: ED (n = 77)	0	0.00
**Time 1: After-Office Hours (*n* = 305)**>		**24,299.34** **(79.67)**
After-Office hours: advice (*n* = 108)	205.17	22,158.36
After-Office hours: referral to clinic (*n* = 17)	125.94	2140.98
After-Office hours: ED (*n* = 180)	0	0.00
**All calls in T1 (*n* = 474)**		**42,224.22** **(89.08)**
**Time 2: Office Hours (*n* = 610)**		**41,644.25** **(68.27)**
Office hours: Advice Given (*n* = 385)	127.43	49,060.55
Office hours: Refer0ral 0to clinic (*n* = 80)	48.2	3856.00
Office hours: ED referral (*n* = 145)	−77.74	−11,272.30
**Time 2: After-Office Hours (*n* = 625)**		**72,749.61** **(116.40)**
After-Office hours: advice (*n* = 311)	205.17	63,807.87
After-Office hours: referral to clinic (*n* = 71)	125.94	8941.74
After-Office hours: ED (*n* = 243)	0	0.00
**All calls in T2 (*n* = 1235)**		**114,393.86** **(92.63)**
**All calls in T1 and T2 (*n* = 1709)**		**$156,618.08** **($91.64)**

^a^ See Additional file 1 for detailed computations of the costs avoided per call

^b^ Mean Cost Avoided per Call = (Cost avoided)/n

## Discussion

Our study showed that telephone consultations to OHNS reduced ED visits in Alberta. Indeed, over 60% of all calls reduced ED visits. Importantly, the majority of these telephone consultations allowed patients to be cared for by their family physicians with < 20% requiring additional outpatient consultation with specialist consults. Moreover, the cost avoidance to the system was substantial despite the procedural changes which relied more on specialists than residents.

Our results are in line with similar studies conducted nationally and internationally. Wilson et al. found British Columbia’s telephone consultation program (Rapid Access to Consultative Expertise) resulted in a 60% prevention of a face-to-face specialist consultation and 32% prevention of ED visits [1]. British Columbia’s pilot project offering only access to cardiologists for family physicians reported that 80% of calls addressed issues adequately through telephone and 20% resulted in further consultation [5]. Wegner et al. in the United States reported that among 306 consults between primary care physicians and pediatric subspecialist, 32% avoided pediatric subspecialist visits; 11% avoided hospital transfers; 5% avoided hospital admissions; and 5% avoided ED visits [6]. In a study in France, Salles et al. reported that 38.3% of 714 calls to geriatricians resulted in advice only and only 4.3% resulted in direct admission to ED, 9.2% in day hospital visit, and 5.3% geriatric consultation [7]. However, Salles reported that 42.9% resulted to planned hospitalization to a geriatrics ward.

The reduction of ED visits was more in T2 than T1, an odds ratio of about 2.5. Various factors could have accounted for this. The volume of calls in T2, which was 2.6 times that of T1, could have provided more opportunities to reduce ED visits. The expertise of the consultants who took the calls in T2 also could have prevented more ED visits when compared to the expertise of the residents. Moreover, as the RAAPID program gained more usage through the years, the calling physicians may have called for decreasing acuities of patients.

With respect to costs, British Columbia’s program reported a cost avoidance of up to $200/call, with an estimated savings of $9005 for 148 calls reviewed, across several specialties [1]. This estimate was based on physicians’ billings for the telephone consultations and estimates of avoided ED visit fees. Our cost estimates model is similar to the RACE program’s cost model, i.e., estimates from direct costs. However, our estimated cost avoidance was smaller at $91.64/call. Our estimates are conservative. Costs from procedures (e.g., endoscopies and laboratory requisitions) and associated hospitalizations would increase the estimate significantly. Indeed, Wegner et al. reported an estimated savings of $477,274 for just 306 consults within the pediatric population [6]. This relatively high estimate represented all associated costs of ED visits and associated costs of hospitalizations, in contrast to our study which only considered physician’s consultation fees and average ED visit costs. Estimates would further be increased with the inclusion of indirect costs from the patient’s perspective, e.g., costs for transportation, food, accommodation, ground or air emergency transportation, and lost time at work for both the patient and accompanying person. One study estimated direct savings from the patient’s expenses, albeit conservatively [3]. For 812 patients, the authors estimated a direct savings of only €1000 for avoidance of travel to EDs, hospitals, and clinics and a savings of €2700 for prevented diagnostic examinations.

Despite our findings, our study is not without limitations. First, this study only evaluated the dispositions at the end of telephone consultations. The events after the calls were not followed through and may not reflect the true disposition. Calls resulting in advice (reducing ED visits), for example, may or may not have resulted in ED visits. Physicians may not comply with all recommendations; however, other studies have shown a high rate of compliance, at 90% [3, 8]. Another study estimates 83% full compliance and a 10% partial compliance [9]. Second, the differences noted between T1 and T2 could be driven by other factors which we were not able to discern. For example, the disproportionately large volume of calls in T2 could represent an increased willingness to use the service in cases which were not as severe compared to when the service initially was launched. And, third, RAAPID policy routed all non-urgent calls received from 2200-0900H to 0900H. As familiarity with the calls increased by the RAAPID center, a disproportionately greater number of calls may have been routed to office hours over time.

## Conclusion

Telephone consultations to OHNS reduced ED visits and specialty consultations in northern Alberta. The telephone consultations facilitated access to OHNS specialists, allowing for patients to be cared for in the community. Collaborative care was delivered while preserving the primary care physicians’ clinical responsibility for the patients.

## Supplementary information

**Additional file 1.**

## Data Availability

Data is available from Alberta Health Services. The authors accessed the data after executing an Alberta Health Services - Data Disclosure Agreement (ID No. RA87657). Fr more information, please email the Corresponding Author.

## Abbreviations

E.D.: Emergency Department
O.H.N.S.: Otolaryngology – Head and Neck Surgery
R.A.A.P.I.D.: Referral, Access, Advice, Placement, Information, and Destination program
R.A.C.E.: Rapid Access to Consultative Expertise
T1: Time 1
T2: Time 2
