# Supplementary material for: Telephone consultations with otolaryngology – head and neck surgery reduced emergency visits and specialty consultations in northern Alberta
Source: J Otolaryngol Head Neck Surg. 2020 Jun 22;49:39. doi: 10.1186/s40463-020-00439-0 (PMC7310048; doi:10.1186/s40463-020-00439-0)
Supplement: Supplementary file 1 — Additional file 1. [file 40463_2020_439_MOESM1_ESM.docx]

Appendix 1 Cost Avoidance Estimates Per RAAPID Call to Otolaryngology – Head and Neck Surgery (OHNS)

|  | (a)  Claim by Referring MD for the call^*^  (CAD) | (b)  Claim by OHNS for the call^**^  (CAD) | (c)  Claim by OHNS for elective specialty clinic^***^  (CAD) | (d)  Average Cost of ED Visit for Diseases  of the Ear-Nose-Throat^****^  (CAD) | (e)  =205.17- (a + b + c + d)  Cost Avoided per Call  (CAD) |
| --- | --- | --- | --- | --- | --- |
| Time 2: Office Hours (calls taken by OHNS Consultants) | | | | | |
| Office hours: Advice given^Note 1^ | 0 | 77.74 | 0 | 0 | $ 127.43 |
| Office hours: Referral to clinic ^Note 2^ | 0 | 77.74 | 79.23 | 0 | 48.2 |
| Office hours: ED referral ^Note 3^ | 0 | 77.74 | 0 | 205.17 | −77.74 |
| Time 2: After-Office Hours (calls taken by OHNS residents) | | | | | |
| After-Office hours: advice ^Note 4^ | 0 | 0 | 0 | 0 | 205.17 |
| After-Office hours: referral to clinic ^Note 5^ | 0 | 0 | 79.23 | 0 | 125.94 |
| After-Office hours: ED ^Note 6^ | 0 | 0 | 0 | 205.17 | 0 |
| Time 1: Office Hours (calls taken by OHNS residents) | | | | | |
| Office hours: advice ^Note 7^ | 0 | 0 | 0 | 0 | 205.17 |
| Office hours: referral to clinic ^Note 8^ | 0 | 0 | 79.23 | 0 | 125.94 |
| Office hours: ED ^Note 9^ | 0 | 0 | 0 | 205.17 | 0 |
| Time 1: After-Office Hours (calls taken by OHNS residents) | | | | | |
| After-Office hours: advice ^Note 10^ | 0 | 0 | 0 | 0 | 205.17 |
| After-Office hours: referral to clinic ^Note 11^ | 0 | 0 | 79.23 | 0 | 125.94 |
| After-Office hours: ED ^Note 12^ | 0 | 0 | 0 | 205.17 | 0 |
| ^*^Referring MD would not bill for the call to the OHNS but for the patient’s consultation.  ^**^Alberta Medical Association Fee Navigator. Health Service Code 03.01LJ (Physician to physician telephone consultations): $ 77.74  ^***^Alberta Medical Association Fee Navigator. Health Service Code 03.08A (Comprehensive consultation): $79.23  ^****^Government of Alberta. Hospital Ambulatory Care Case Costs - 2017 version | | | | | |

Formula:


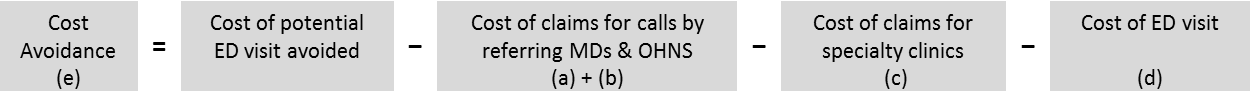


where,

Cost of Potential ED Visit Avoided = $205.17

Cost of claims for calls by referring MD = $0. It is assumed that referring MDs would already have billed for the patient’s consultation and would not bill for the telephone call.

Cost of claims for calls by OHNS: $0 if taken by residents; $77.74 if taken by consultants.

Cost of claims for specialty clinics: $0 if the disposition after the calls did not entail a referral to a specialty clinic; $79.23 if the disposition after the calls entailed a referral to a clinic.

Cost of ED visits: $0 if the disposition after the calls did not entail an ED visit; $205.17 if the disposition after the calls entailed an ED visit.

Possible Scenarios.

Assumption. Had there been no RAAPID consultation, the patient would have been sent to the ED and a cost of $205.17 incurred by the healthcare system.

If the RAAPID consultations resulted in a disposition of being given advice, the healthcare system would have avoided the cost of an ED visit ($205.17) minus the cost of claims from the calls.


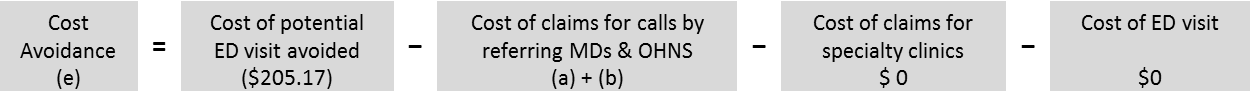


If the RAAPID consultations resulted in a disposition of being referred to a specialty clinic, the healthcare system would have avoided the cost of an ED visit ($205.17) minus the cost of claims from the calls and minus the claims for the specialty clinic.


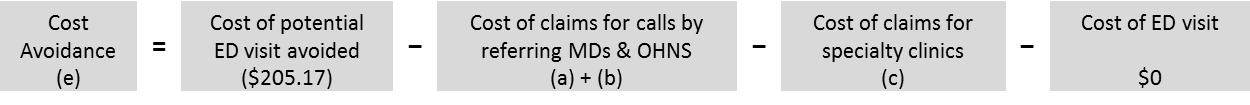


If the RAAPID consultations resulted in a disposition of being referred to the ED, the potential avoided cost of an ED visit is cancelled by the referral for an ED visit. Moreover, the healthcare system would have incurred the cost of claims from calls.


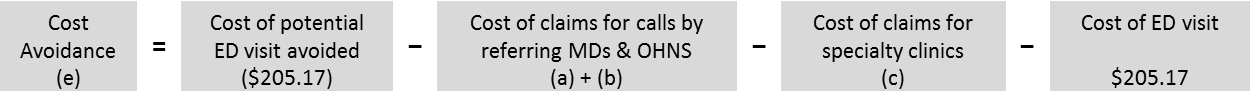


Notes

1. For time 2 during office hours: cost avoidance when the disposition was advice given
2. Claim by Referring MD for the Call: 0
3. Claim by receiving OHNS Consultant for the Call: $77.74
4. Claim by receiving OHNS for elective specialty clinic: 0

Rationale: There would be no elective specialty clinic because only advice was given.

1. Average Cost of ED Visit for Diseases of the Ear-Nose-Throat: 0

Rationale: There would be no ED visit because only advice was given.


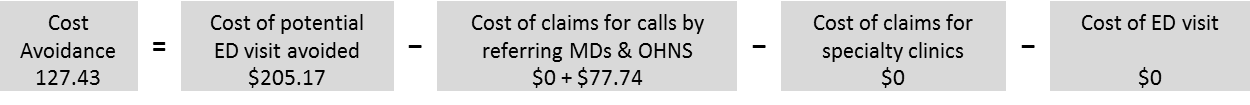


1. For time 2 during office hours: cost avoidance when the disposition was referral to clinic
2. Claim by Referring MD for the Call: 0
3. Claim by receiving OHNS Consultant for the Call: $77.74
4. Claim by receiving OHNS for elective specialty clinic: $79.23
5. Average Cost of ED Visit for Diseases of the Ear-Nose-Throat: $0

Rationale: There would be no ED visit because of the referral to the clinic.


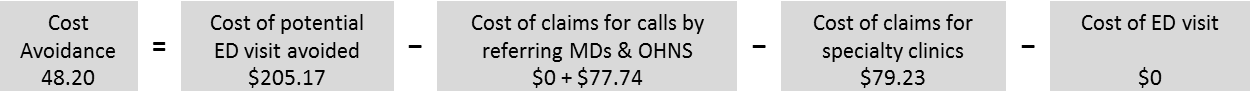


1. For time 2 during office hours: cost avoidance when the disposition was ED referral
2. Claim by Referring MD for the Call: 0
3. Claim by receiving OHNS Consultant for the Call: $77.74
4. Claim by receiving OHNS for elective specialty clinic: $0

Rationale: No referral to specialty clinic.

1. Average Cost of ED Visit for Diseases of the Ear-Nose-Throat: $205.17


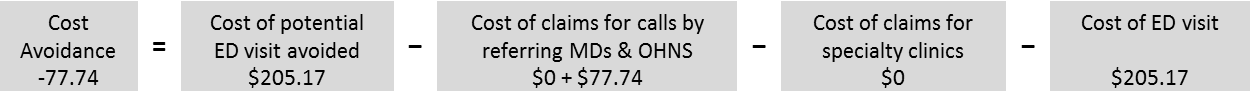


1. For time 2 after office hours: cost avoidance when the disposition was advice given
2. Claim by Referring MD for the Call: 0
3. Claim by receiving OHNS Resident for the Call: $0

Rationale. The consultants did not claim for calls taken by residents.

1. Claim by receiving OHNS for elective specialty clinic: 0

Rationale: There would be no elective specialty clinic because only advice was given.

1. Average Cost of ED Visit for Diseases of the Ear-Nose-Throat: 0

Rationale: There would be no ED visit because only advice was given.


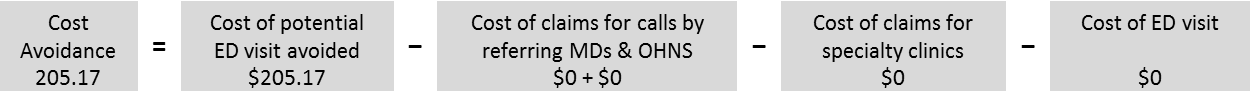


1. For time 2 after office hours: cost avoidance when the disposition was referral to clinic
2. Claim by Referring MD for the Call: 0
3. Claim by receiving OHNS Resident for the Call: $0
4. Claim by receiving OHNS for elective specialty clinic: $79.23
5. Average Cost of ED Visit for Diseases of the Ear-Nose-Throat: $0

Rationale: There would be no ED visit because of the referral to the clinic.


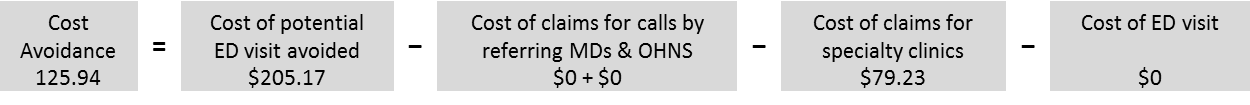


1. For time 2 after office hours: cost avoidance when the disposition was ED referral
2. Claim by Referring MD for the Call: 0
3. Claim by receiving OHNS Resident for the Call: $0
4. Claim by receiving OHNS for elective specialty clinic: $0

Rationale: No referral to specialty clinic.

1. Average Cost of ED Visit for Diseases of the Ear-Nose-Throat: $205.17


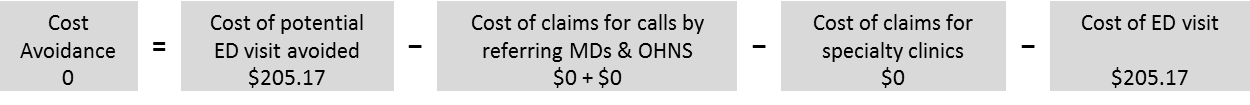


1. For time 1 during office hours: cost avoidance when the disposition was advice given

Same as in Note 4.

1. For time 1 during office hours: cost avoidance when the disposition was referral to clinic

Same as in Note 5.

1. For time 1 during office hours: cost avoidance when the disposition was ED referral

Same as in Note 6.

1. For time 1 after office hours: cost avoidance when the disposition was advice given

Same as in Note 4.

1. For time 1 after office hours: cost avoidance when the disposition was referral to clinic

Same as in Note 5.

1. For time 1 after office hours: cost avoidance when the disposition was ED referral

Same as in Note 6.

References

Alberta Medical Association. Fee Navigator [Internet]. Edmonton: Alberta Medical Association. 2019 [cited 2019 Feb 17]. Available from: <https://www.albertadoctors.org/fee-navigator>

Government of Alberta. Health Costing – Hospital Ambulatory Care Case Costs [Internet]. Edmonton: Alberta Government [cited 2019 February 20]. Available from: <https://open.alberta.ca/opendata/health-costing-hospital-ambulatory-care-case-costs>
